# A Simple Bone Cyst in Cervical Vertebrae of an Adolescent Patient

**DOI:** 10.1155/2017/8908216

**Published:** 2017-05-28

**Authors:** Adriana Bruges Boude, Lina González Vásquez, Fernando Alvarado-Gomez, María Constanza Bedoya, Andrés Rodríguez-Múnera, Luis Carlos Morales-Saenz

**Affiliations:** Department of Orthopedic Surgery, Fundación Santa Fe de Bogotá, Bogotá, Colombia

## Abstract

**Introduction:**

Spinal simple bone cysts, also known as solitary cysts, are extremely unusual benign primary bone tumors with few cases reported in the literature.

**Case Presentation:**

Incidental Magnetic Resonance Imaging (MRI) finding of a C2 Simple bone cyst in a 13-year-old female patient is reported. Complementary studies suggested the benign nature of the lesion. Patient underwent cervical curettage followed by tumor excision. A lateral submandibular approach to the upper cervical spine was used and careful bone resection was possible with a radiofrequency assisted burr and no instrumentation or fixation was required. The stability of the defect was ensured by filling it with bone allograft and by prescribing a postsurgical plastic cervical collar to maintain neck immobilization. Histological examination supported the diagnosis of simple bone cyst. At 6–12-month follow-up the patient presented no recurrence or symptomatology.

**Conclusions:**

Solitary bone cysts are infrequent entities in the cervical vertebrae and preservation of spine stability without instrumentation to avoid neurological complications is often challenging. In this case, the proximity of the cyst to the right vertebral artery and the risk of injury were high; however the surgical approach used was successful and no recurrence or instability was evidenced on postoperative MRI.

## 1. Introduction

Primary bone lesions in the spine have an annual global prevalence of 2.5 to 8.5 cases for every 100.000 persons and its distribution varies significantly according to age [1]. Spinal tumors represent about the 2–8% of all skeletal muscle tumors in children and most of them are benign. In order of frequency, eosinophilic granulomas present in 12–25% of cases and osteoid osteoma/osteoblastoma in 12% and aneurysmal bone cysts represent a 10% of the cases [2, 3].

Among primary bone tumors, solitary bone cysts are infrequent lesions that occur mostly in males with a ratio of 2.5 : 1 [4]. This type of tumor is found primarily in long bones, 80% of the cases in the proximal humerus followed by the distal femur [5]. Rarely, they affect the vertebral spine bones, but when they occur, unspecific and persistent neck pain is found and most cases are considered incidental findings. Simple bone cysts affecting the spine are extremely unusual and very few have been reported in the literature specially in children [6, 7]. We present a simple bone cyst in the vertebral body of C2 found incidentally in a 13-year-old teenager.

## 2. Case Presentation

**Figure 1:**
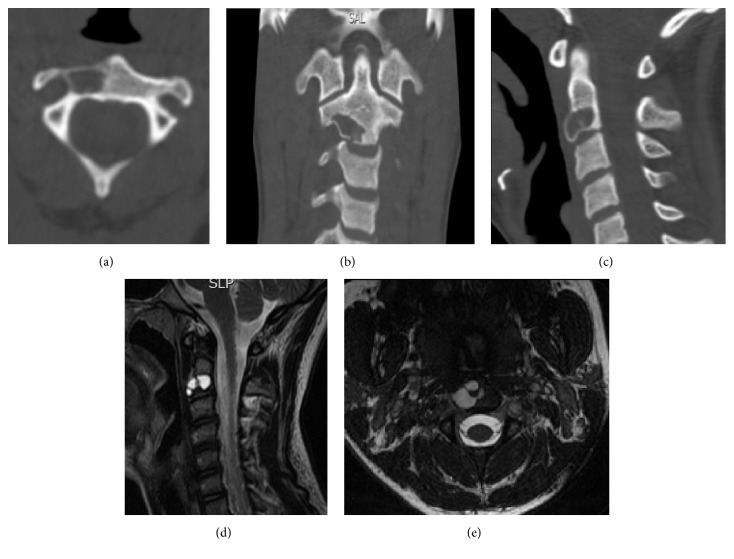
(a), (b), and (c) CT Lesion with cystic appearance, defined borders compromising two-thirds of body C2, in close proximity to the right vertebral artery. (d) MRI of the lesion. (e) Axial sequence of the preoperative MRI.

A 13-year-old female was admitted to hospital for acute demyelinating polyneuropathy confirmed by electromyography and nerve conduction studies; later on a diagnosis of Guillain Barré Syndrome was confirmed. A complementary MRI performed as part of in-hospital management showed an incidental finding of a cystic lesion in the vertebral body of C2 (Figure 1). The only symptom reported by the patient was cervical pain irradiated to shoulders. The physical exam was unremarkable, and no deformities nor neurologic alterations were noted. The patient started using a Philadelphia collar and restriction of exercise was recommended.

**Figure 2:**
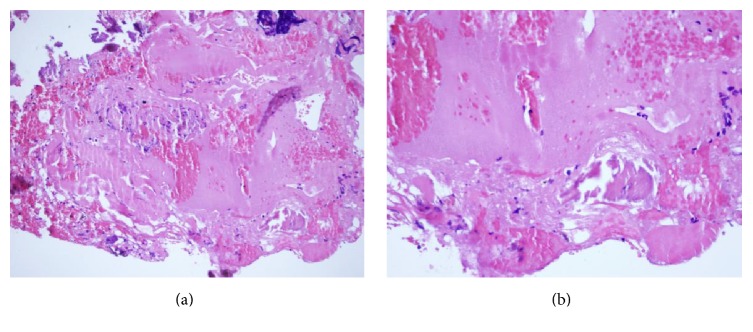
(a) The first microphotograph shows in the inferior part coated by a thin membrane and abundant eosinophilic deposits, hemorrhagic material hematoxylin and eosin, magnification 20x. (b) 40x magnification of the microphotograph.

The case was discussed at the neurosurgery, spine surgery, and neuroradiology service meeting and extension studies such as angiotomography of carotid arteries and vertebrobasilar system and bone scintigraphy were requested to determine the etiology of the lesion because the management and prognosis vary according to the type of tumor. Studies concluded it was a tumoral lesion with benign characteristics. Additionally, correlation with computed tomography (CT scan) revealed a focal hypodense lesion in the right half of the vertebral body of C2 with disruption of the inferior cortical. Because of the elevated risk of fracture and collapse of the vertebra due to the thinning of the cortical the optimal treatment considered was surgical resection of the tumor to prevent further neurological lesions. Cervical curettage followed by tumor excision was performed. A lateral submandibular approach to the upper cervical spine was used and bone resection was performed with a radiofrequency assisted burr, no instrumentation or fixation was required. Intraoperatively a cystic lesion with fibrotic and nodular material was evidenced; later histologic examination ruled out giant cell tumor and aneurismatic bone cyst (Figure 2). The stability of the defect was ensured by filling it with cortical/cancellous allograft and by prescribing a postsurgical plastic cervical collar to maintain neck immobilization.

**Figure 3:**
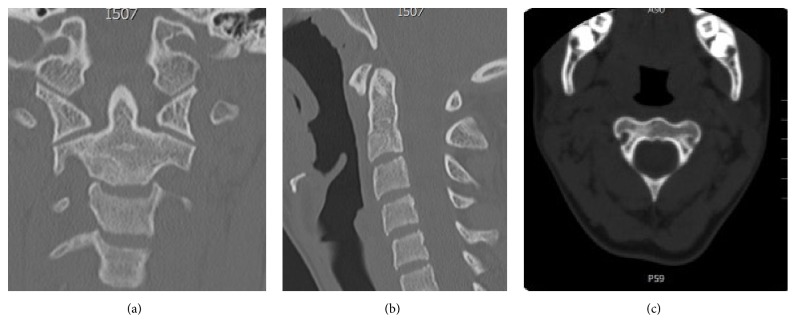
(a), (b) Postoperative CT scan that shows no recurrence and adequate integration of the graft. (c) An image corresponding to postoperative sagittal CT.

Postoperative evolution was satisfactory. The patient remained with a CTLSO immobilization for 4 months. At 6 and 12 months, the patient presented no symptoms and the postoperative CT scan showed adequate integration of the graft as shown in Figure 3.

## 3. Discussion

In younger population the differential diagnosis must include aneurismal bone cysts, which in this case was the first diagnostic possibility considered in our patient, in second place giant cell tumor and less likely simple bone cysts. Aneurismal cysts are osteolytic multiloculated lesions, filled with fluid, predominant in women in the third decade of life and affect long bone metaphysis. In the CT scan the lytic lesions can be seen with expansion and thinning of the bone cortex. In the MRI the multiloculated appearance with septum is notorious [4, 8].

In our case this pattern was not evidenced in the MRI. Giant cell tumors are lytic benign primary bone tumors that show a local aggressive behavior. They are commonly found among people between 20 and 40 years old specially in long bones and represent 5% of the tumors [1]. Three factors did not support this diagnostic possibility: first our patient was a teenager which does not fit the demographics of this tumor, second, no evidence of local aggressive behavior of the tumor was observed, and lastly the tumor in our patient was located in the vertebral body of c2 contrasting the most common presentation of giant cell tumors (most commonly in the sacrum followed by the thoracic and lumbar spine). Additionally, giant cell tumors appear in the CT scan as lytic lesions with thickness loss and destructive expansion of the sacrum or vertebrae. In a MRI the classic presentation is a multiloculate mass with blood in the interior [4]. These imaging patterns were not consistent with the imaging findings in our patient. In the case presented it was necessary to perform several imaging studies to characterize the tumor because this has implications in the management planning of these cases. A more infrequent differential diagnosis is simple bone cysts which are considered after ruling out the aforementioned diagnostic possibilities. Histological evidence supported the simple bone cyst diagnosis. Solitary bone cysts are uncommon entities in the cervical spine; they occur specially in older adults and have been described as the only true cysts with primary intraosseous origin [9]. Only a limited number of cases have been found in the cervical spine [7]. These benign tumors are common in long bone metaphysis like the proximal humerus, femur, and tibia [4, 6, 10]. Ogata et al. reported simple bone cyst cases managed by surgical curettage and use of bone graft without recurrence [9]. We undertook the same surgical approach in this patient and evidenced no recurrence at 6 and 12 months.

The cause of solitary bone cysts is still unknown; some authors have proposed a posttraumatic or posthemorrhagic etiology which could explain the vertebral location especially among the elderly. However, our patient did not have history of trauma or bleeding. In children it has been suggested that developmental defects in the epiphyseal plate can originate these tumors, but in this case the unusual location of the cyst points towards ruling out this etiology.

There is still no agreement regarding the best therapeutic approach [7, 9, 11]. Because of the high rate of recurrence in young patients, surgery is not always the election treatment [10], but it might be considered when they present in rare anatomic locations such as the spine since their rates of recurrence are lower. In the case reported, the lesion involved the upper cervical spine and the thinning of the cortex made the bone prone to fractures producing instability or migration of bone components to the spinal canal leading to severe neurological damage. The surgical approach followed here was successful, and 12 months after the procedure, the patient has not developed recurrent lesions or instability.

The uncommon presentation of these lesions in the cervical spine makes their diagnosis challenging. In cases were histological samples are inconclusive, imaging studies become critical in the diagnostic process and planning of the surgical approach. Other aspects to take into account are the age and daily life demands of the patient. Our patient was a young teenager who practices sports and her daily physical demands augmented the risk of fracture and collapse of the vertebra. In consequence we decided to treat her lesion by curettage and allograft to preserve stability and prevent the risk of fracture with further migration of the fractured bone tissue into the spinal cavity with fatal consequences for the patient

In conclusion solitary bone cysts are infrequent entities in the cervical vertebrae and preservation of spine stability without instrumentation to avoid neurological complications is often challenging. In this case, the proximity of the cyst to the right vertebral artery and the risk of injury was high; however the surgical approach used was successful and no recurrence or instability was evidenced on postoperative MRI.
